# Retrieval Deficiency in Brain Activity of Working Memory in Amnesic Mild Cognitive Impairment Patients: A Brain Event-Related Potentials Study

**DOI:** 10.3389/fnagi.2016.00054

**Published:** 2016-03-23

**Authors:** Bin-Yin Li, Hui-Dong Tang, Sheng-Di Chen

**Affiliations:** ^1^Department of Neurology and Collaborative Innovation Center for Brain Science, Rui Jin Hospital Affiliated to Shanghai Jiao Tong University School of MedicineShanghai, China; ^2^Laboratory of Neurodegenerative Diseases and Key Laboratory of Stem Cell Biology, Institute of Health Science, Shanghai Institutes for Biological Sciences, Chinese Academy of Science and Shanghai Jiao Tong University School of MedicineShanghai, China

**Keywords:** event-related potentials, amnesic mild cognitive impairment, working memory, retrieval, sLORETA

## Abstract

In the early stage of Alzheimer disease (AD) or mild cognitive impairment (MCI), working memory (WM) deficiency is prominent and could be attributed to failure in encoding, maintenance or retrieval of information. However, evidence for a retention or retrieval deficit remains equivocal. It is also unclear what cognitive mechanism in WM is impaired in MCI or early AD. We enrolled 46 subjects from our Memory Clinics and community, with 24 amnesic MCI patients and 22 normal subjects. After neurological and cognitive assessments, they performed a classic delayed match to sample (DMS) task with simultaneous event-related potential (ERP) recorded. The ERPs in encoding and retrieval epoch during WM were analyzed separately. The latency and amplitude of every ERP component were compared between two groups, and then analyzed to explore their relationship with neuropsychological performance. Finally, the locations of maximal difference in cortex were calculated by standard low-resolution tomographic analysis. A total of five components were found: P1, N1, P2, N2, and P300. The amplitude of P2 and P300 was larger in normal subjects than in MCI patients only during retrieval, not encoding epoch, while the latency did not show statistical difference. The latency and amplitude of P1 and N1 were similar in two groups. P2 amplitude in the retrieval epoch positively correlated with memory test (auditory verbal learning test) and visual spatial score of Chinese Addenbrooke's Cognitive Examination-Revised (ACE-R), while P300 amplitude correlated with ACE-R. The activation difference in P2 time range was maximal at medial frontal gyrus. However, the difference in cortex activation during P300 time range did not show significance. The amplitude of P2 indicated deficiency in memory retrieval process, potentially due to dysfunction of central executive in WM model. Regarding the location of P2 during WM task, medial frontal plays important role in memory retrieval. The findings in the present study suggested that MCI patients have retrieval deficit, probably due to central executive based on medial frontal gyrus. Thus, it may provide new biomarker for early detection and intervention for aMCI.

## Introduction

Cognitive decline is usually considered as an age-related phenomenon, which involves a continuous disease course from preclinical state, mild cognitive impairment (MCI) to Alzheimer's disease (AD). Prevalence of MCI in population-based epidemiological studies ranges from 3 to 20% in adults older than 60 or 65 years old (Gauthier et al., 2006; Ravaglia et al., 2008; Jia et al., 2014; Ding et al., 2015). Some MCI patients remain stable or even return to normal over time (Gauthier et al., 2006), while approximately half patients will progress to AD over 4–5 years.

Early detection and intervention for AD is remarkably important. Initially, most researchers agreed that the early and predominant cognitive deficit in AD is episodic memory impairment. However, cognitive processes under this kind of memory and their deficit in early AD were unclear in long period (Germano and Kinsella, 2005). Working memory (WM) provides a new insight into memory theory, which is defined as the capacity to hold information that is absent in mind for brief periods of time (Baddeley, 2012) and is closely related with daily activity performance.

In WM model, it involves at least three processes: information encoding, maintenance, and retrieval. All information-processing steps are regulated and controlled by the central executive. The central executive system is able to allocate attention to concurrent tasks, against potentially distracting irrelevant information. Long-term memory is formed when information is transferred from temporary or short-term storage to permanent episodic representation by rehearsal (Baddeley, 2001).

In early AD, memory complaint could be attributed to encoding, maintenance, and retrieval of information. The inability to encode and retrieve memory is present at the earliest stages of the disease and deteriorates during disease course (Aggarwal et al., 2005). Which part is the most problematic one? It is generally observed that a deficit in the acquisition of new information (encoding) is characteristic of early AD (Pasquier et al., 2001), while evidence for a retention or retrieval deficit in early AD remains equivocal (Grober and Kawas, 1997; Albert et al., 2001). However, all these observation is based on behavioral tests. As poor memory performance could be resulted from any deficit in the information processing, it is still unclear which cognitive mechanism is impaired in AD.

As WM works as a serial process at first a few seconds, event-related potentials (ERPs) provide a high temporal-resolution method to compare time-related cognitive process between different populations. Pinal et al. (2014) assessed the time course of brain activity during encoding and retrieval. Larger N2 amplitude and stronger activation of the left temporal lobe were found after long maintenance periods during information retrieval.

They also (Pinal et al., 2015a) used ERP recording and a delayed match to sample (DMS) task to assess age-related changes in young and old adults during successful information encoding in WM. The result revealed that smaller P2 and P300 amplitudes may signal the existence of an age dependent reduction in the processing resources available, and P2 and N2 latencies were longer in old than in young subjects.

In similar DMS, younger individuals have more posterior regions involved in WM processing, while adulthood has more anterior regions involved (Barriga-Paulino et al., 2015). Functional MRI (fMRI) study provided high spatial-resolution evidence. Galashan et al. (2015) combined EEG and fMRI with the same task, and they found a temporal pattern of source activities starting in occipital and temporal brain regions, followed by a simultaneous engagement of parietal and frontal brain regions and a later activity of a source in pre-supplementary motor area.

To our knowledge, no studies have compared the difference of cognitive process in WM between MCI and normal aging people. The encoding and retrieval deficit in WM also remains controversial in MCI patients. Amnesic MCI (aMCI) is one important type of pre-AD cognitive state with or without disability in other cognitive domains. WM studies in MCI patients revealed increased P200-N200 latencies, as well as an early dysfunction of neural generators within the parietal cortex in two-back paradigm (Missonnier et al., 2005, 2007). However, these studies did not uncover the deficit in information processing of WM. Therefore, we recruited aMCI patients from our memory clinics, and used ERP technique to precisely record time course of brain activity during encoding and retrieval in WM task.

## Materials and methods

### Subjects

We recruited normal elderly subjects from community and patients with memory complaint for more than 6 months from our Memory Clinics of the Department of Neurology, Rui Jin Hospital from December 2014 to July 2015. All participants were firstly evaluated by memory-disorders specialists and screened by the Mini Mental State Examination (MMSE, Chinese Version; Katzman et al., 1988), Zung Self-rating Anxiety Scale (SAS) and Zung Self-rating Depression scale (SDS). Each patient from memory clinics was subsequently performed blood test for possible causes of memory impairment including thyroid function, syphilis, HIV, folic acid, and vitamin B12, etc. Brain MRI scanning was also done for detecting vascular causes and hippocampus atrophy. Finally, the diagnosis of MCI was based on a detailed medical history, relevant physical and neurological examinations, negative laboratory findings, and neuroimaging studies. Exclusion criteria included evidence of stroke, Parkinson's disease, HIV/AIDS, mood problems, and reversible dementias, as well as treatment with benzodiazepines, antipsychotic, or antiepileptic medications. Patients with poor vision were also excluded.

A total of 46 subjects were finally enrolled. A patient was diagnosed as MCI patients if any of the following criteria were met as proposed by Petersen et al. (1999): memory has become worse gradually, as reported by the subject or his/her caregiver; objective evidence of impaired memory compared with normal controls matched for age, gender, and education; Clinical Dementia Rating global score = 0.5; normal activities of daily living, and not demented. Finally, 24 patients were diagnosed as MCI and 22 subjects from community as normal control (CDR = 0 and normal activities of daily living) by two neurologists independently (Li and Chen; Table 1).

**Table 1:** **Demographic data and task performance (Mean ± Standard Deviation)**.

	**MCI (*n* = 24)**	**Normal control (*n* = 22)**	***P***
Age	69.27±7.55	69.17±8.91	0.947
Sex (female/all)^*^	9∕24	8∕22	0.936
Year of education^#^	13.33±2.80	14.18±3.09	0.206
MMSE^#^	26.41±2.12	28.95±0.95	< 0.001
Reaction time (ms)^#^	1166.53±160.33	1079.63±141.08	0.068
Accuracy (%)^#^	68.58±12.39	75.77±11.37	0.090
ACE-R^##^	84.50±7.33	93.26±3.61	< 0.001
AVLT-Immediate^##^	5.04±1.13	6.87±1.33	< 0.001
AVLT-20 min^##^	2.81±2.14	6.78±2.68	< 0.001
Digit-Symbol^##^	35.12±10.20	45.44±8.55	0.003
CFT-Copy^##^	34.75±1.48	35.38±1.24	0.181
CFT-recall^##^	11.56±8.62	19.53±8.22	0.011
SCWT C/A^##^	3.38±0.80	3.19±1.10	0.566
STT-A^##^	68.36±20.07	52.67±17.76	0.020
STT-B^##^	168.73±43.65	120.43±38.57	0.001

* Chi-square test,

# Mann-Whitney U-test,

## The total sample size is 35, with 16 subjects in MCI group and 19 subjects in control group.

Independent t-test were performed. ACE-R, Addenbrooke's Cognitive Examination-Revised; AVLT, Auditory Verbal Learning Test-Huashan version; STT, Shape Trail Test (including Part A and B); CFT, the Rey-Osterrieth Complex Figure Test (CFT); SCWT, Stroop Color-Word Test.

### Neuropsychological assessment

A neuropsychological battery for multiple cognitive domains was performed in 35 subjects among 46 subjects by trained researcher who was blind to diagnosis (16 MCI patients and 19 normal subjects). These tests included the Chinese Addenbrooke's Cognitive Examination-Revised (ACE-R) (Fang et al., 2014), the Auditory Verbal Learning Test (AVLT)-Huashan version (Zhao et al., 2012), the Shape Trail Test (STT, including Part A and B), the Rey-Osterrieth Complex Figure Test (CFT), the Stroop Color-Word Test (SCWT), and the Symbol-Digit Modalities Test (SDMT).

Our study received ethical approval from the Research Ethics Committee, Rui Jin Hospital Affiliated to Shanghai Jiao Tong University School of Medicine, Shanghai, China. Written informed consent was obtained from all subjects or their guardians. All subjects were of unrelated Chinese Han descent with education level of secondary school or above, and knew English letter very well.

### The delayed match to sample task

**Figure 1:**
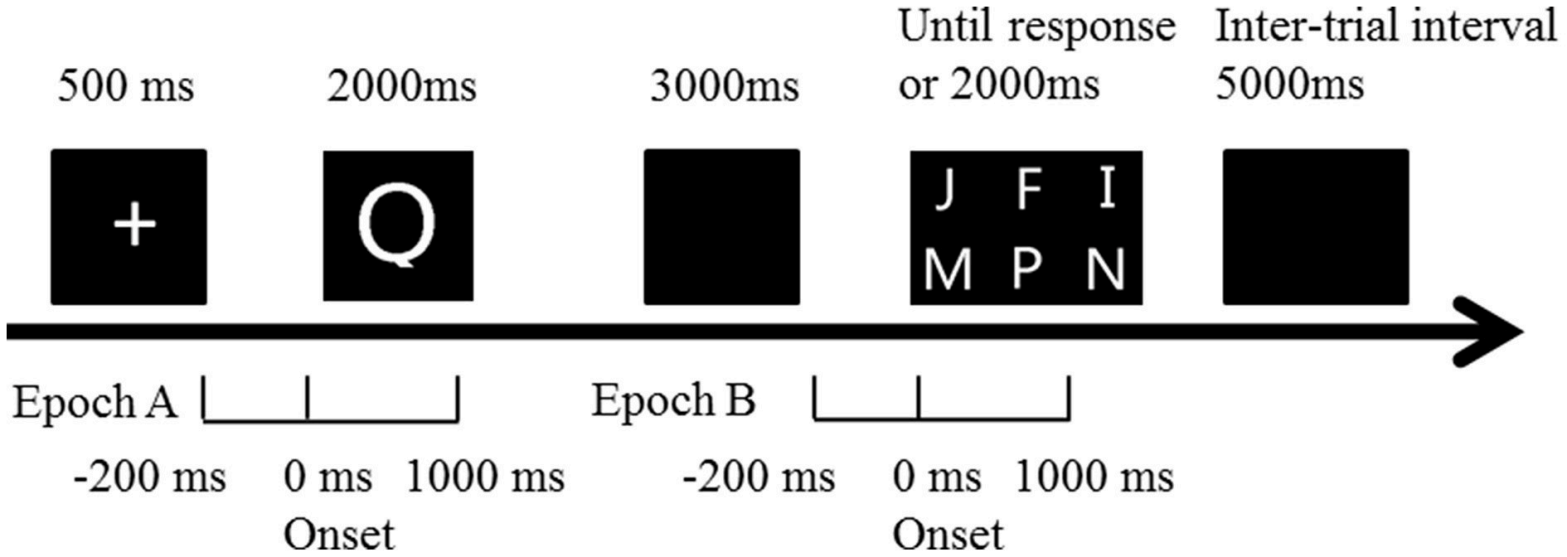
**Illustration of the trial time course in the DMS paradigm**. Subjects were presented with one single letter (sample stimulus). They had to keep it in mind for a fixed delay (3 s) before judging as quickly and accurately as possible whether it was presented among the presenting six letters (probe stimulus). ERP epoch A (encoding period): extends from 200 ms prior to sample stimulus onset to 1000 ms after. ERP epoch B (retrieval period): extends from 200 ms prior to probe stimulus onset to 1000 ms after.

Subjects first took a simple English letter test to make sure everyone knew all 26 letters. Then every subject was asked to complete a DMS task, which employed a visual task with WM. All visual stimuli were white presented on a dark background. In one trial, after a “+” in the middle of screen for 500 ms, one single letters (sample stimulus) randomly selected from 26 letters were displayed in the center of screen for 2000 ms. Subject was asked to keep it in mind. A blank followed the letter lasted for 3000 ms as an interruption. Probe stimulus (six letters randomly selected from 26 letters in a 2 ^*^ 3 matrix) was then presented in the middle of screen (Figure 1). Subject has to judge whether the letter seen before was among the six letters (press numeric keyboard “1” for “yes,” “2,” for “no”). The matrix was presented until the subject pressed key or for 2000 ms at maximum. After 5000 ms blank for a rest, a new trial started. Every individual underwent two sequential blocks. One contained 20 trials for practice, and the other contained 100 trials for test. In 50% of trials in each block, the correct answer was “yes,” while the left was “no.” “YES” and “No” trials were randomized in each block. Not until the subject had accuracy over 50% in practice block could she or he start the test block. The task was programmed and behavioral data (reaction time and accuracy) was recorded by E-Prime 2.0 software (Psychology Software Tools, Inc., Pittsburgh, PA, USA).

Memory storage of the first letters was required in order to compare it with the probe stimuli. The stimuli were large (height of 5.5° visual angle), bright (55 cd/m2), and presented in the middle of a computer monitor in order to make it easy for the subjects to see them. The whole test lasted for about 15–18 min.

### EEG recordings and data processing

EEG and electro-oculogram (EOG) were recorded with a 32-channel EEG amplifier (BrainAmp by Brain Products, Munich, Germany) using 32 Ag-AgCl electrodes. EEG electrodes were set in a standard EEG cap according to extended 10–20 system with inter-optode distances between 2 and 3 cm. Thirty-two scalp electrodes (Fp1, Fp2, F3, F4, C3, C4, P3, P4, O1, O2, F7, F8, T7, T8, P7, P8, Fz, FCz, Cz, Pz, FC1, FC2, CP1, CP2, FC5, FC6, CP5, CP6, FT9, FT10, TP9, TP10) with reference to bilateral linked ear lobes (TP9, TP10) recorded electrical brain activity while the subject performed the DMS task.

The sampling rate of every channel was 500 Hz, with frequency band-pass from 0.1 to 70 Hz. Artifact criteria were applied to all electrodes. Recorded data were passed through a digital filter with the high cut-off frequency set at 50 Hz and with a low cut-off frequency set at 0.1 Hz. Notch-filter centered at 50 Hz was also applied to minimize electrical line noise. Ocular artifacts were corrected using the Infomax Restricted algorithm in an Independent Component Analysis as implemented in Brain Vision Analyzer 2.0 (Brain Products GmbH). Artifact rejection was followed by semi-automatic check (maximal allowed voltage step: 50 μV/ms; maximal allowed difference of values in intervals: 200 μV; lowest allowed activity in intervals: 0.5 μV). Mean artifact rejection rate for all MCI subjects was >1%. Then data was segmented in two epochs (Figure 1). Encoding epoch lasted from 200 ms before presentation of sample stimulus to 1000 ms after, while retrieval epoch lasted from 200 ms before probe stimulus to 1000 ms after. Baseline correction was done with the mean activity in the 200 ms prior to stimulus, and ERPs were based on correct trials.

### Statistical analysis

#### ERP component analysis

Based on the grand-averaged ERP waveforms, five ERP components were searched both in encoding and retrieval, according to the reports reviewed in the introduction section: N1, P1, N2, P2, and P300. We chose electrodes and then calculated peak latency and amplitude on the basis of studies with the same paradigm, as well as choosing the electrodes where amplitude was maximal. The N1 peak was measured as the voltage at the largest negative going peak in the latency window of 150–210 ms after stimulus onset at P7 and P8. The P1 was at the maximum peak in the latency window of 84–140 ms at O1, O2, P7 and P8. The N2 was considered at the most negative peak between 230 and 300 ms at F3, F4, C3, C4, CZ, FZ, FCz, while P2 was between 150 and 250 ms at FC1, FC1, CZ, FZ, FCz. P300 was identified as the maximum positive peak at O1, O1, and Pz between 250 and 450 ms after stimulus onset.

### Demographics, psychometrics and ERPs

The difference of age, education level, MMSE score, reaction time, accuracy and sex between two groups was compared by the Mann-Whitney or Pearson's chi-square test. Reaction time (RT) was defined as the time between the onset of probe stimulus and subject's response, only in the correct trials. ERP components' parameters (latency and amplitude in each component) were compared by independent *t*-test between two groups in both encoding and retrieval epochs. Any statistically significant findings in the comparison lead to deep analysis for behavioral and electrophysiological association. The potential relationship between neuropsychological performance and brain activity was estimated by correlation analysis and linear regression. Pearson's correlation was calculated separately between each cognitive assessment score and ERP components parameters.

### Standard low resolution tomography analysis (s-LORETA)

The sLORETA is a functional imaging method for locating current source and widely used in EPR studies (Pandey et al., 2012). The LORETA algorithm has been identified as an efficient tool for functional mapping, also in encoding or retrieval period of WM studies (Pinal et al., 2015a). We employed sLORETA version that is identical with the one proved validated in previous studies (Pandey et al., 2012), available at: http://www.uzh.ch/keyinst/loreta.htm.

As described in sLORETA manual, a collection of volume elements in brain cortex has been modeled in the digitized Montreal Neurological Institute (MNI) coordinates corrected to the Talairach coordinates. In the study, the averaged waveforms (500 time samples, 1000 ms, post-stimulus) from 32 original recording montages for each subject were converted and saved into ASCII values. Transformation matrix was also constructed from these original data and electrode coordinates. The sLORETA values of each subject were computed for separately using ASCII values, electrode coordinates, and the transformation matrix. Regarding the sLORETA analysis, it compared differences between two groups in the time range of specific waveforms which showed difference in amplitude or latency, and constructed images to localize these differences in the three dimensional (3-D) space within the brain. For each comparison, one single test (Log of ratio of averages) was calculated for time-samples in significant ERP components (based on results from ERPs analysis above) with 5000 random permutations. The resultant values were then plotted in a 3-D brain model and evaluated for the level of significances. We also reported the maximum differences between groups at respective MNI coordinates and Brodmann areas (BA).

## Results

### Demographics and task performance

The age, education years and sex proportion were similar in two groups. The independent Mann-Whitney *U*-test of task performance data (accuracy and RT) revealed marginally significant difference (*p* = 0.090 and 0.068), with a bit lower accuracy and longer reaction time in MCI patients. The MMSE score in MCI group was much lower than control group, consistent with their global cognitive ability and our clinical diagnosis. The matched age and education years in two groups helped to balance cognitive-related factors in the following analysis. Neuropsychological assessment also provided critical difference in cognitive tasks (AVLT, SDMT, CFT) where memory played a major role (Table 1).

### Brain activity in encoding and retrieval

**Figure 2:**
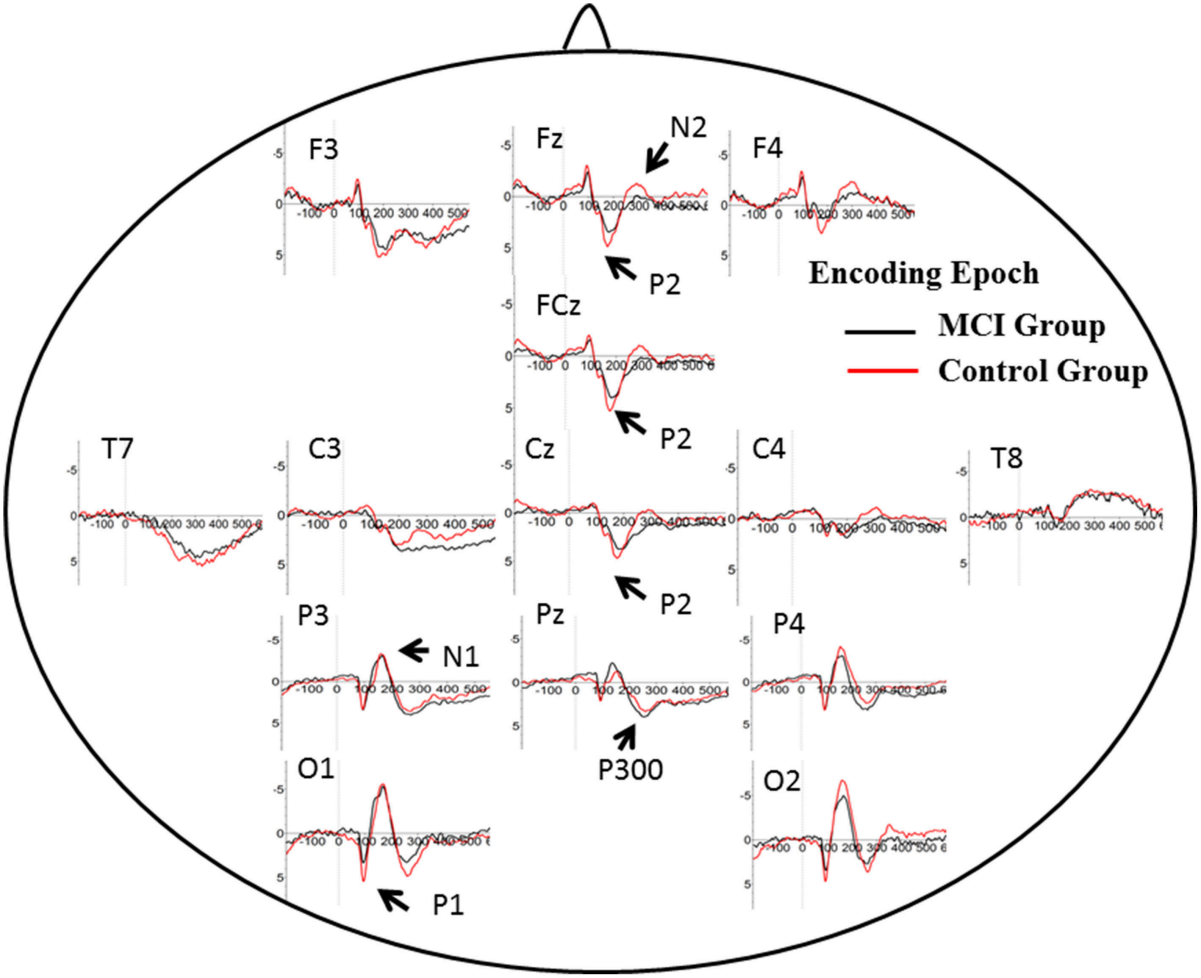
**Grand average ERP waveform in aMCI (black line) and normal control group (red line) during retrieval period in WM task**. Time was shown in milliseconds where stimulus onset was at 0, and potentials were shown in micro-voltage.

**Figure 3:**
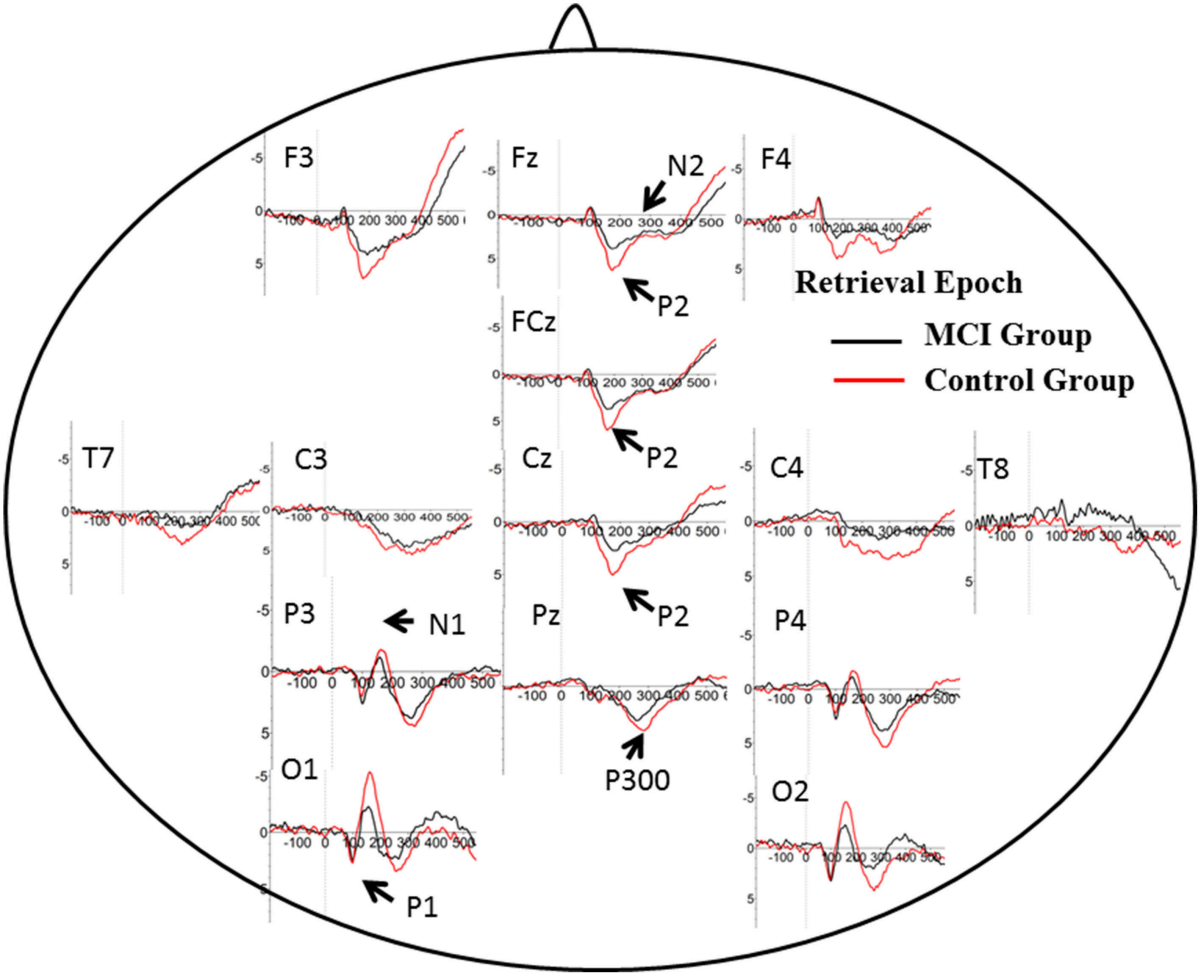
**Grand average ERP waveform in aMCI (black line) and normal control group (red line) during encoding period in WM task**. Time was shown in milliseconds where stimulus onset was at 0, and potentials were shown in micro-voltage.

The number of valid trials used for averaging ERPs in both encoding and retrieval epoch did not differ between two groups (Tables 2, 3). In the encoding epoch, no significant effect of group was found in parameters of all five ERPs components (Figure 2, Table 2). In the retrieval epoch (Figure 3), the independent *t*-test revealed significant effect of group in the amplitude of P2 and P300 components (*p* = 0.025 and 0.038). The P2 and P300 amplitude in aMCI group was smaller than in control group. However, latency of those two components was not significantly affected by the group effect. Regarding the N1, N2, and P1, neither difference in amplitude or latency reached statistically significant between two groups (Table 3).

**Table 2:** **Parameters of ERP component in encoding epoch (Mean ± Standard deviation)**.

**Encoding**		**MCI (*n* = 24)**	**Normal control (*n* = 22)**	p^*^
Number of valid trials		1643	1637	0.889
P300	Latency	317.69±46.51	312.64±45.17	0.711
	Amplitude	4.37±2.66	4.50±2.88	0.803
P2	Latency	191.65±20.77	182.69±16.59	0.115
	Amplitude	5.02±2.04	5.77±3.22	0.344
N2	Latency	272.55±18.12	269.58±14.36	0.543
	Amplitude	−0.29±2.15	−1.21±2.27	0.164
P1	Latency	79.72±6.38	80.63±8.40	0.676
	Amplitude	4.72±2.65	5.06±2.07	0.638
N1	Latency	173.12±12.08	173.45±12.51	0.928
	Amplitude	−6.27±3.27	−7.21±3.24	0.335

Latency and amplitude were measured in milliseconds (ms) and micro-voltage (μV).

* Independent T-test.

**Table 3:** **Parameters of ERP component in retrieval epoch (Mean ± Standard deviation)**.

**Retrieval**		**MCI (*n* = 24)**	**Normal control (*n* = 22)**	p^*^
Number of valid trials		1642	1604	0.634
P300	Latency	296.67±37.75	306.54±34.80	0.363
	Amplitude	3.72±2.31	5.45±3.14	***0.038***
P2	Latency	195.30±28.33	183.87±21.42	0.165
	Amplitude	4.83±2.62	6.81±3.14	***0.025***
N2	Latency	267.25±19.26	267.58±16.92	0.951
	Amplitude	0.70±2.62	1.21±2.84	0.531
P1	Latency	85.05±7.28	82.91±7.79	0.341
	Amplitude	4.44±2.88	4.01±2.47	0.597
N1	Latency	167.08±14.57	170.08±10.96	0.396
	Amplitude	−4.41±3.52	−5.96±3.13	0.123

Latency and amplitude were measured in milliseconds (ms) and micro-voltage (μV).

* Independent T-test. Bold and italic values are indicated as statistical significance.

### Brain activity and cognitive ability

**Figure 4:**
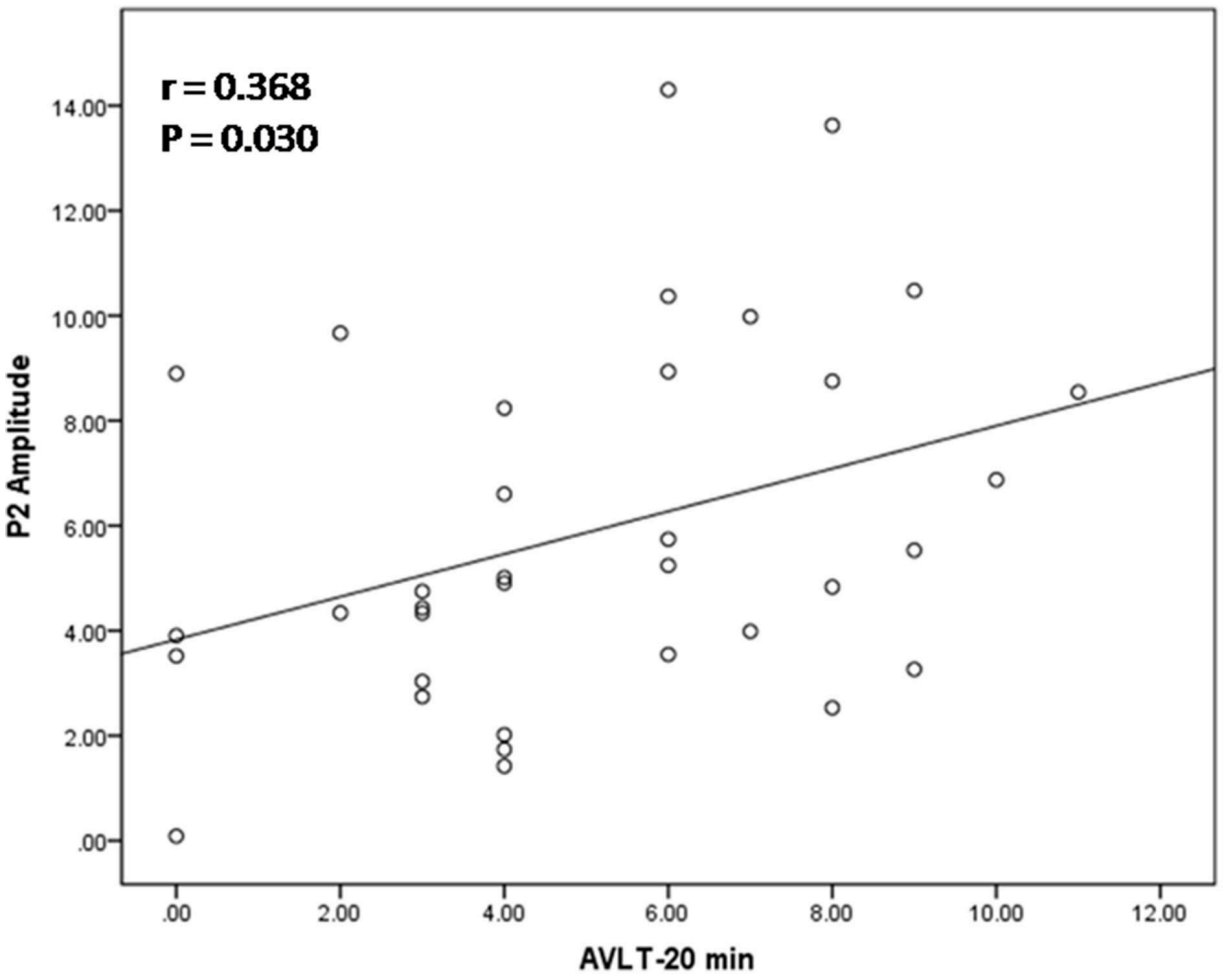
**Relation between P2 amplitude and score of auditory verbal learning test (recall after 20 min)**. The scatterplot and best fit line (*r* = 0.368 and *p* = 0.030) referred to Pearson's correlation analysis.

Among all subjects, a neuropsychological battery for multiple cognitive domains was performed in 35 subjects (16 MCI patients and 19 normal subjects). Based on the results of ERP components analysis, the Pearson's correlation was calculated in all subjects between cognitive assessment score and ERP components parameters (the amplitude of P2 and P300) separately. Regarding P2 amplitude, significant correlation was found in its relationship with AVLT-20 min recall (*r* = 0.368, *p* = 0.030) and scores of visual spatial ability in ACE-R. Scores in AVLT-5 min recall and recognition were marginally significant correlated with P2 amplitude (*r* = 0.286, *p* = 0.096; *r* = 0.292, *p* = 0.089). The amplitude of P300 was correlated with ACE-R (*r* = 0.395, *p* = 0.019), as well as scores of language fluency and visual spatial ability in ACE-R (*r* = 0.374, *p* = 0.027; *r* = 0.363, *p* = 0.032). A scatterplot was depicted to show the fit line and relationship between P2 amplitude and AVLT-20 min recall (Figure 4).

### Standardized low resolution tomography analysis (sLORETA)

**Figure 5:**
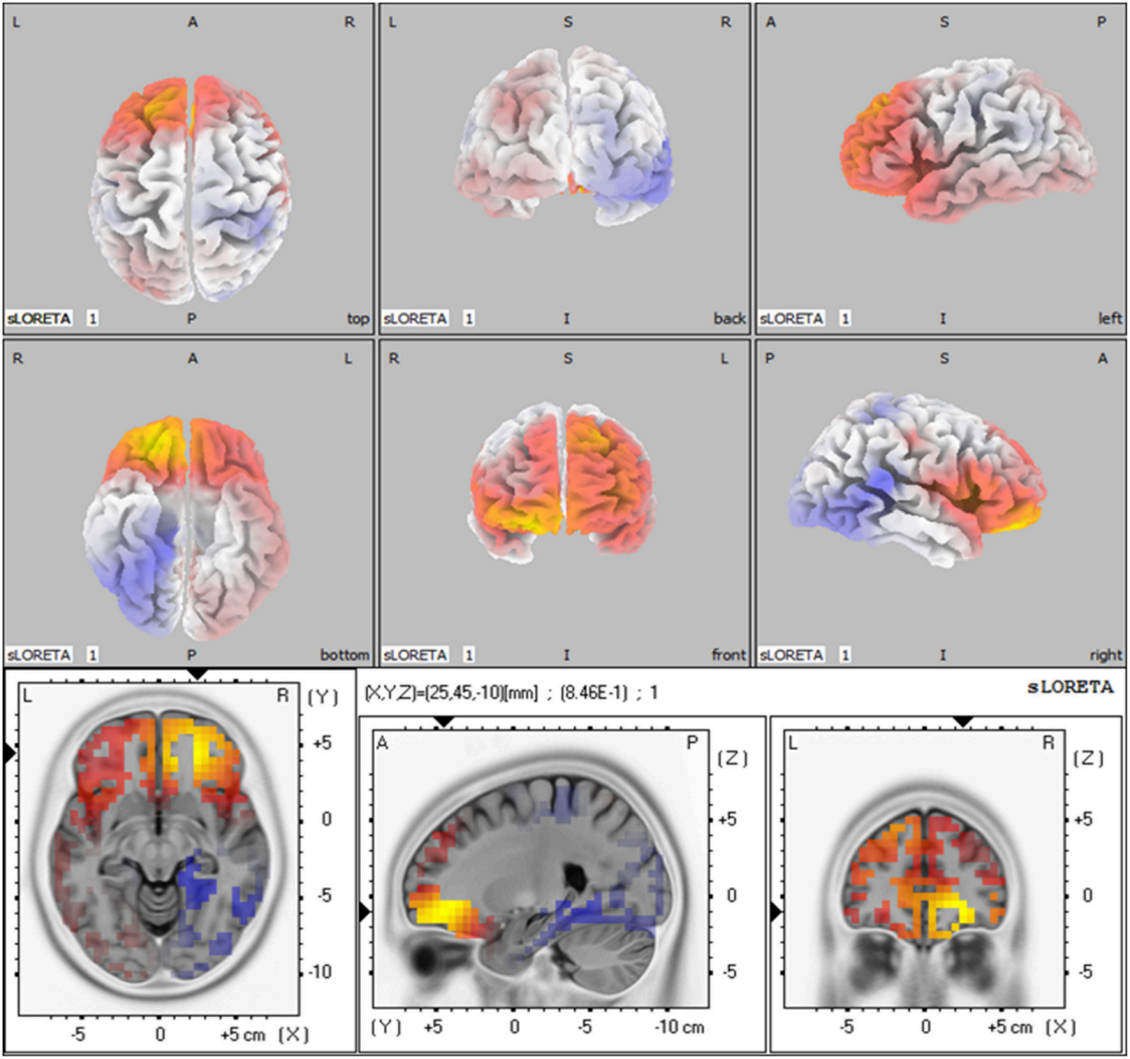
**The sLORETA images showing statistical differences between aMCI and control group (3D-view and slice-view) in the P2 time-range**. The three slice-view images below located the maximal difference between aMCI group and control group (MNI coordinates *x, y, z* = 25, 45, −10). Positive difference was in yellow color with reference of MCI group.

Based on the results showed above, we performed statistical non-parametric mapping (SnPM) during P2 and P300 time range (in the retrieval epoch) to show different activation in these specific temporal intervals. The statistical difference between groups for current density at the source is shown in Table 4. The brain areas that show activation differences at < 0.05 level are shown in Figure 5 (positive in yellow color, while negative in blue color with reference of MCI group). As shown in Table 4, the difference in P2 time range was maximum at BA 11 (medial frontal gyrus) of the right frontal cortex (Log of ratio of averages = 0.846, *p* < 0.01). Besides, BA 10 (superior frontal gyrus) also showed markedly activated in control group during P2 time range. However, the difference in cortex activation during P300 time range did not reach significant level.

**Table 4:** **Comparison of aMCI and normal control group in sLORETA (voxels showing maximal difference)**.

**MNI coordinates**			**Brodmann area**	**Brain region**	**Log of ratio of average**	***p*-Value**
***X***	***y***	***Z***				
**P2 TIME-RANGE DURING RETRIEVAL (150–250 ms)**						
25	45	−10	11	Frontal lobe, Medial Frontal Gyrus	0.845	< 0.05
25	45	−5	11	Frontal lobe, Medial Frontal Gyrus	0.845	< 0.05
20	45	−5	10	Frontal lobe, Superior Frontal Gyrus	0.832	< 0.05
**P300 TIME-RANGE DURING RETRIEVAL (250–450 ms)**						
−5	55	−25	11	Frontal lobe, Rectal Gyrus	0.512	>0.1

MNI, Montreal Neurological Institute.

## Discussion

### Working memory task performance and ERPs

It is shown that in the MCI and AD patients, longer maintenance intervals hampered the task performance. Patients performed significantly worse than controls on the associative WM task (van Geldorp et al., 2015). In our study, the two groups had similar age, sex proportion and education level. Meanwhile, the interval between two stimuli was fixed and relatively short (Pinal et al., 2015b). Although aMCI patients did have poor cognitive ability in psychometric tests, the results showed similar WM task performance (reaction time and latency) in two groups. However, the brain activity measured as ERPs still uncovered difference under seemingly comparable WM performance. It suggested that brain activity deficit underlie behavioral change and the electrophysiological test provided opportunity of early detection and intervention.

### Brain activity and neuropsychological function

It is still unclear whether encoding or retrieval is impaired in MCI patients. In this exploratory study, the ERPs in encoding and retrieval epoch were separately analyzed. Some ERP components proved sensitive to probe stimulus. It is noted that the findings in ERPs made no sense until it could be related with real cognitive ability. Every ERP should be analyzed with the consideration of behavioral and cognitive ability.

P2: P2 has been related to various cognitive process: a semantic effect (Peressotti et al., 2012), top–down mechanism for rapid evaluation of stimulus significance (Paynter et al., 2009) or subjectively relevant stimuli (Schapkin et al., 2000; Potts and Tucker, 2001). Larger amplitude for P2 was observed in the control group than in MCI group. In WM model, retrieval requires phonological loop and central executive, which is generally defined as attention allocation efficiency (Germano and Kinsella, 2005; Wang et al., 2010). Previous studies concerning aging effects on P2 amplitude showed larger P2 amplitude in younger adults, suggesting deficit in the allocation of processing resources for the evaluation of stimulus significance. Consistently, positive correlation was found between the degree of improvement in WM capacity and the post-pre difference in P2 amplitudes after WM training (Li et al., 2015). However, the latency of P2 in the present study did not differ in two groups. It suggested little slowing in stimulus judgment during information encoding and retrieval in WM.

In DMS task, subjects had to search probe stimulus (a 2^*^3 letter matrix) for the sample, indicating involvement of attention allocation in the retrieval. Besides, the P2 amplitude found in retrieval epoch positively correlated with memory recall test (AVLT-20 min recall). These results supported the role of P2 in information retrieval, as well as regulation of attention allocation.

The P2 amplitude also showed correlation with visual-spatial score of ACE-R. The fact may give explanation that all subjects were not English native speakers and they largely recognize English letter via its appearance.

P300: P300 is widely studied in cognitive processes, and represented different psychological meaning under different tasks (Kirschner et al., 2015). Previous studies have found an age-related reduction in the amplitude of P300 component at parietal electrodes, suggesting lower amounts of processing resources available for allocation to stimulus categorization (McEvoy et al., 2001; Saliasi et al., 2013; Pinal et al., 2015a). However, the difference existed in encoding epoch, while our study showed difference of P300 amplitude in retrieval epoch when age was balanced between groups. Positive correlation was found between P300 amplitude and memory load, indicating its significance in modulating resources for stimulus classification and context updating (Pinal et al., 2014). As memory load was same in present two groups, difference in P300 suggested that aMCI patients could modulate fewer resources and thus evoked lower amplitude. Similar to P2, the latency of P300 in two groups did not differ from each other, suggesting relative spared processing speed in aMCI patients. The P300 amplitude also showed correlation with score of ACE-R, especially in language fluency and visual-spatial ability. It suggested that P300 might represent more general cognition ability, compared with P2.

N2: N2 (predominant in anterior part of brain) has been studied in correlation with visual information encoding (Nittono et al., 2007). Consistently, N2 waveform was obvious in anterior electrodes in the present study. Previous studies found positive association between N2 latency and task accuracy or N2 amplitude with memory load (Pinal et al., 2014, 2015a). Stimulus discrimination and classification may be reflected by N2 (Folstein and Van Petten, 2008), hence higher N2 amplitude happens when the stimulus includes more information load. There were no difference between aMCI and control group for N2 amplitude and latency. Low and fixed memory load (only one letter) in the present may give an explanation.

P1 and N1: It is generally accepted that these early ERP components usually represent perceptual processing of visual inputs (Vogel and Luck, 2000; Taylor, 2002). The present results indicate that the deficit in information retrieval does not affect perceptual processing of stimuli, which is also consist with DMS study in young and older subjects (Pinal et al., 2015a).

### Medial frontal lobe in working memory

In the present study, MCI patients had less activated brain area in medial frontal area than control subject during P2 time range in WM retrieval. As MRI has high spatial resolution than ERPs, fMRI provides more precise activated location.

At retrieval, “retrieval-success network” was found in brain areas which is thought to be content-independent (Wagner et al., 2005; Huijbers et al., 2010). These areas found in fMRI studies include the posterior midline region consisting of the precuneus, retrosplenial cortex, and posterior cingulate, the medial prefrontal cortex as well as the hippocampus.

In a fMRI study with DMS task, correct decision at WM probe was linked to activation in the anterior and posterior midline brain structures (Bergmann et al., 2015). The result was generally consisted with our findings in sLORETA. As ERP provided higher time-resolution, frontal midline structures in 150–250 ms were less activated in aMCI group. Animal experiment provided evidence that shifting of attention set in the rat impaired with lesions of prefrontal cortex, and medial frontal cortex associated with impairment in initial acquisition and reversal learning.

As discussed above, P2 correlated with attention allocation in the retrieval. Locations found in sLORETA further verified its neuro-anatomical basis and functional significance.

One limitation of the present study is a bit fewer channel data (32-channel data) in sLORETA analysis. Though our findings in sLORETA was generally consistent with fMRI study, 64 or 128-channel EEG cap in further studies could provide more detailed and accurate information.

## Conclusion

The aim of the present study was to explore brain activity changes in MCI patients during WM task. We tried to find clinically significant electrophysiological waveform for WM and its location in the cortex. The results revealed that brain activity during retrieval process of WM was impaired in aMCI patients, while encoding process spared. In retrieval process, the amplitude of P2 indicated deficient function of central executive in the WM model. Besides, medial and superior frontal gyrus markedly activated during P2 time range in the control group. The findings in the present study suggested WM retrieval deficit in aMCI patients, probably due to dysfunction of central executive (attention allocation) based on medial and superior frontal gyrus. It may provide new biomarker for early detection and intervention for aMCI.

## Author contributions

All three authors cooperated and contributed to the design and plan of the present study. BL was in charge of ERP data acquisition, analysis and manuscript writing. HT was in charge of neuropsychological assessments and analysis. SC was in charge of clinical diagnosis and manuscript verifying.

### Conflict of interest statement

The authors declare that the research was conducted in the absence of any commercial or financial relationships that could be construed as a potential conflict of interest.
